# Psycholinguistic dataset on language use in 1145 novels published in English and Dutch

**DOI:** 10.1016/j.dib.2020.106655

**Published:** 2020-12-16

**Authors:** Severi Luoto, Andreas van Cranenburgh

**Affiliations:** a English, Drama and Writing Studies, University of Auckland, 1010 Auckland, New Zealand; b School of Psychology, University of Auckland, 1010 Auckland, New Zealand; c Department of Information Science, University of Groningen, Oude Kijk in 't Jatstraat 26, 9712 EK Groningen, the Netherlands

**Keywords:** Stylometry, Literature, LIWC, Psycholinguistics, Corpus linguistics, Digital humanities, Sex, Sexual orientation

## Abstract

This dataset includes psycholinguistic data on 694 English-language and 451 Dutch-language novels, acquired with computerised analysis of digitised novels published mainly between 1800 and 2018. The English-language novels have a total word count of 66.9 million words, while the Dutch-language novels comprise 49.6 million words, therefore offering large, representative samples for both languages. The data provided in this article include 93 linguistic and psycholinguistic outcome variables for the English-language novels, acquired using Linguistic Inquiry and Word Count (LIWC) version 2015, and 68 linguistic and psycholinguistic outcome variables for the Dutch-language novels, acquired using Linguistic Inquiry and Word Count (LIWC) version 2001. The dataset also includes word frequencies (unigram and bigram) for each novel. The metadata for each novel include year of publication, authors’ nationality, sex, age at publication, and sexual orientation (the latter only in the English-language dataset), making it possible for researchers to study the data along these parameters. The use of these data can help researchers illuminate how word use reflects psychological processes in more than two centuries of literary art in English and in contemporary Dutch novels.

## Specifications Table

**Table:** 

Subject	Social Sciences and Humanities
Specific subject area	Linguistics, Psychology, Digital Humanities
Type of data	Table
How data were acquired	The data were extracted from digitised versions of novels using Linguistic Inquiry and Word Count (LIWC) versions 2015 and 2001, and a Python script to count word frequencies.
Data format	Raw
Parameters for data collection	Novelists for the English set were identified using literary anthologies, literary award nominees and winners, biographical guides, and online lists of LGBT writers. Novels for the Dutch sets were collected using bestseller lists and literary award nominees and winners.
Description of data collection	Digitised versions of the novels were extracted from various online and offline sources. All novels were cleaned manually of prefaces, introductions, content tables, postscripts, biographical notes, author notes, footnotes, and publishers’ additional commercial material included at the end of many novels to prevent them from affecting the data analyses. For the English-language novels, authors’ sexual orientation was recorded using biographical information, including information on the sex of any partners (married or otherwise) that the authors had or any self-identification related to sexual orientation that the authors may have made publicly known.
Data source location	English texts: • http://www.gutenberg.org/ • http://gutenberg.net.au/ • https://archive.org/ • https://www.library.auckland.ac.nz/ • https://www.aucklandlibraries.govt.nz/ • http://digital.library.upenn.edu
	Dutch texts: • Commercially available ebooks • Commercially available printed books • Electronic texts shared by publishers
Data accessibility	http://dx.doi.org/10.17632/tmp32v54ss.2

## Value of the Data

• Computerised text analysis using LIWC data can help researchers illuminate how language use reflects psychological processes in more than two centuries of literary art.
• The dataset can be useful for psychologists, linguists, literary scholars, and other social scientists working on the psychology of language.
• These data can help researchers address questions related to linguistics, psychology of language, language change, fiction, authors’ sex, and sexual orientation.
• This dataset provides psycholinguistic data on canonical and prizewinning novels in English and Dutch, as well as canonical and less well-known novels by sexual minority writers.
• The data are based on a large set of texts comprising 116.5 million words, which enables researchers to tap into large-scale psycholinguistic data.
• The metadata on the English-language corpus include year of publication, authors’ nationality, sex, sexual orientation, and age at publication of each novel, making it possible for researchers to study the data along these parameters. The metadata on the Dutch-language corpora include year of publication, authors’ nationality, sex, and age at publication of each novel, novels’ original language, and the novels’ genre category.

## 1 Data Description

This dataset includes psycholinguistic data on a corpus of 694 English-language novels (total word count: 66.9 million words) and 451 Dutch-language novels (total word count: 49.6 million words). The 100 000 most frequent unigrams and bigrams for each novel are also included. The psycholinguistic data have been derived from electronic versions of the novels using Linguistic Inquiry and Word Count (LIWC) versions 2015 (for the English-language novels) and 2001 (for the Dutch-language novels).

The novelists included in these samples were selected using literary anthologies [1], [2], [3], [4], [5], biographical guides [6], [7], [8], [9], online lists of LGBT writers [10], [11], [12], bestseller lists [13], and literary awards [14,15]. The English-language novels were published mainly between 1800 and 2018 (*M* = 1959.94, *SD* = 54.136).^1^ The Dutch-language novels were published mainly in the 21st century (*M* = 2009.76, *SD* = 1.977).

The English-language sample of novels by heterosexual authors includes canonical works such as James Joyce's *Ulysses*, Jane Austen's *Sense and Sensibility*, and Herman Melville's *Moby Dick*, as well as works by contemporary bestselling authors such as Ian McEwan and Kazuo Ishiguro. Pulitzer Prize winners and National Book Award winners are included in the English-language sample from 1965 to 2018 subject to availability of electronic versions of their novels. Booker Prize winners and finalists and Pulitzer prize finalists from 1969 to 2018 are also included in the English-language sample subject to availability of their novels. The homosexual samples include classics such as John Rechy's *City of Night* from 1963 and Radclyffe Hall's *The Well of Loneliness* from 1928. The homosexual and bisexual samples include many novels from authors who may be less well known: the sampling protocol for homosexual and bisexual authors was not based on literary prizewinners or finalists, because it was difficult (if not impossible) to obtain large samples that way.

The LIWC data on the English-language novels are included in the file english_metadata_and_liwc.csv, available in the Supplementary Material (http://dx.doi.org/10.17632/tmp32v54ss.2). Each of the output variables from LIWC is written as one column of data to an output file. Each text file (i.e. novel) is written as a row. The first 13 columns include metadata such as Novel ID, Author ID, authors’ sex, sexual orientation, name, nationality, year of birth, publication year, and author's age when each novel was published. The subsequent columns present the output data from LIWC2015, from ‘segment’, ‘word count’, and ‘analytical thinking’ through to ‘other punctuation’. ‘Segment’ has the value “1” for each novel because each novel was analysed as a whole text instead of dividing the text into smaller segments. For more details on the LIWC2015 variables reported in the English-language dataset, readers may refer to [16,17]. The novels (i.e. rows) in the English-language LIWC file are organised according to authors’ sex and sexual orientation, starting from heterosexual males, heterosexual females, homosexual males, and homosexual females through to bisexual females. Bisexual male authors were not included in the sample because of the paucity of authors who could be identified as such.

The Dutch-language samples consist of two sets of novels. The Riddle corpus [13] contains 401 novels selected based on being bestsellers in the period 2009–2012; both original Dutch novels as well as novels translated into Dutch are included. The Nominees corpus [14,15] consists of 50 novels by Dutch and Flemish authors nominated for either the AKO Literatuurprijs (shortlist) or the Libris Literatuur Prijs (longlist) in 2007–2012. The LIWC data on the Riddle corpus is included in the file named dutch_riddle_metadata_and_liwc.csv, while the LIWC data on the Nominees corpus can be accessed in the file dutch_nominees_metadata_and_liwc.csv, both available in the Supplementary Material. ‘Segment’ has the value “1” for each novel because each novel was analysed as a whole text instead of dividing the text into smaller parts. For more details on the LIWC2001 variables reported in the Dutch-language dataset, readers may refer to [18,19].

We also extracted unigram and bigram word frequencies from the texts (i.e., bag-of-word features). Unigrams are individual word counts, while bigrams are counts for pairs of consecutive words. The word frequency data of the English-language sample are available in the file named english_ngrams.zip, while the word frequency data for the Dutch-language Riddle and Nominees corpora can be accessed using the files named dutch_riddle_ngrams.zip and dutch_nominees_ngrams.zip, respectively, all available in the Supplementary Material. The n-gram files are in CSV format and consist of document-term matrices with novels as rows and terms as columns; the respective cells for each combination of novel and term contain the corresponding counts. The columns are ordered by frequency and restricted to the 100 000 most frequent terms.

**Fig. 1:**
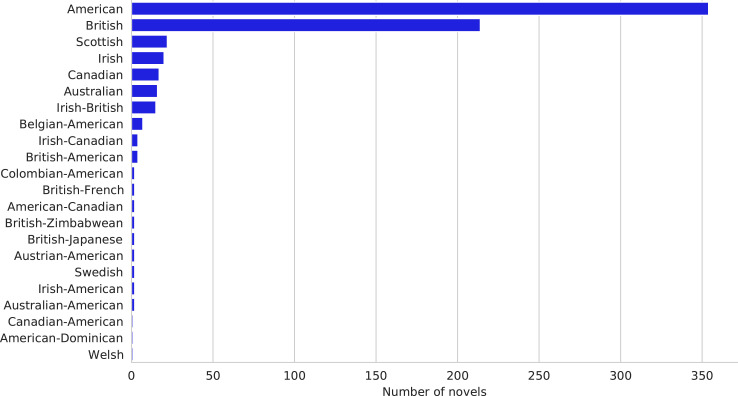
Nationalities of the authors in the English-language dataset. American (*n* = 354 novels) and British (*n* = 214 novels) authors form the majority of the sample, while 126 novels were written by authors of other nationalities.

**Fig. 2:**
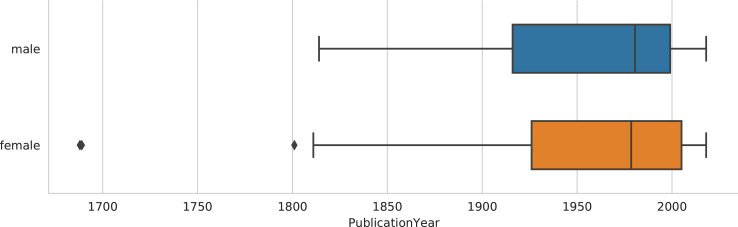
Distribution of publication year partitioned by authors’ sex in the English-language sample (*n* = 694 novels). Medians are shown as vertical lines inside the boxes. Box = interquartile range (25%–75%); whiskers = nonoutlier range; diamond = outlier. The only novels published before the 19th century were those by Aphra Behn, a bisexual female author whose three novels included in this dataset were published in 1688–1689.

**Fig. 3:**
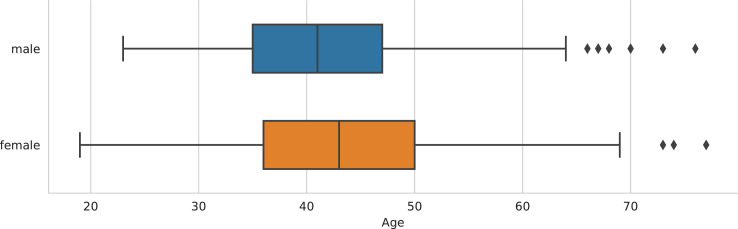
Authors’ age at publication partitioned by authors’ sex in the English-language sample (*n* = 694 novels). Medians are shown as vertical lines inside the boxes. Box = interquartile range (25%–75%); whiskers = nonoutlier range; diamond = outlier.

**Fig. 4:**
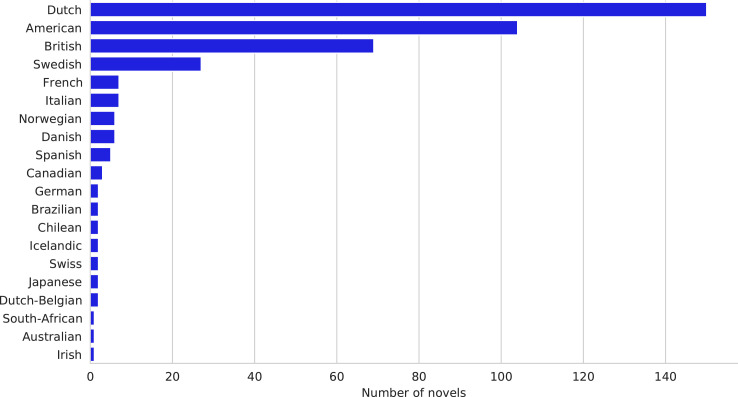
Nationalities of the authors in the Dutch-language Riddle dataset (*n* = 401 novels). In this dataset, 152 novels were originally written in Dutch and 249 novels were translated into Dutch from other languages.

**Fig. 5:**
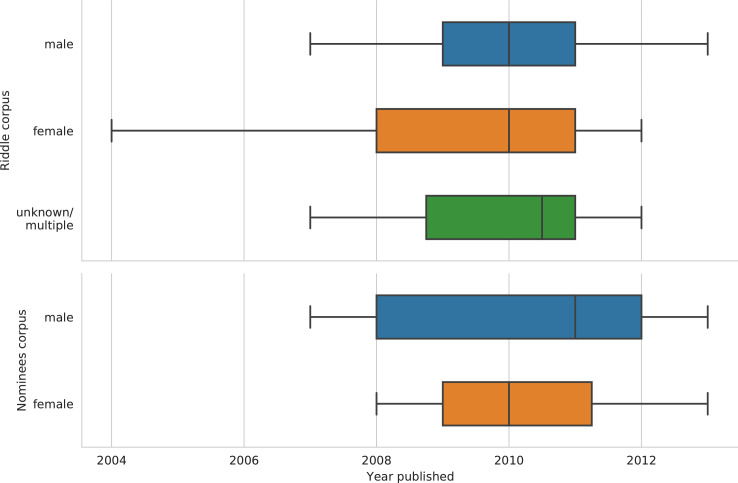
Distribution of publication year partitioned by authors’ sex in the Dutch-language Riddle dataset (*n*_males_ = 191 novels; *n*_females_ = 196 novels; *n*_unknown/multiple_ = 14 novels), and the Nominees dataset (*n*_males_ = 26 novels; *n*_females_ = 24 novels). Medians are shown as vertical lines inside the boxes. Box = interquartile range (25%–75%); whiskers = nonoutlier range.

**Fig. 6:**
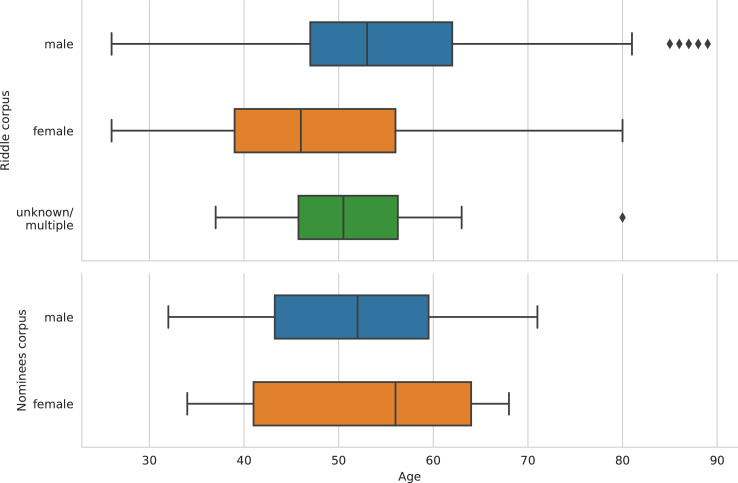
Authors’ age at publication partitioned by authors’ sex in the Dutch-language Riddle corpus (*n* = 401 novels) and Nominees corpus (*n* = 50 novels). Medians are shown as vertical lines inside the boxes. Box = interquartile range (25%–75%); whiskers = nonoutlier range; diamond = outlier.

**Fig. 7:**
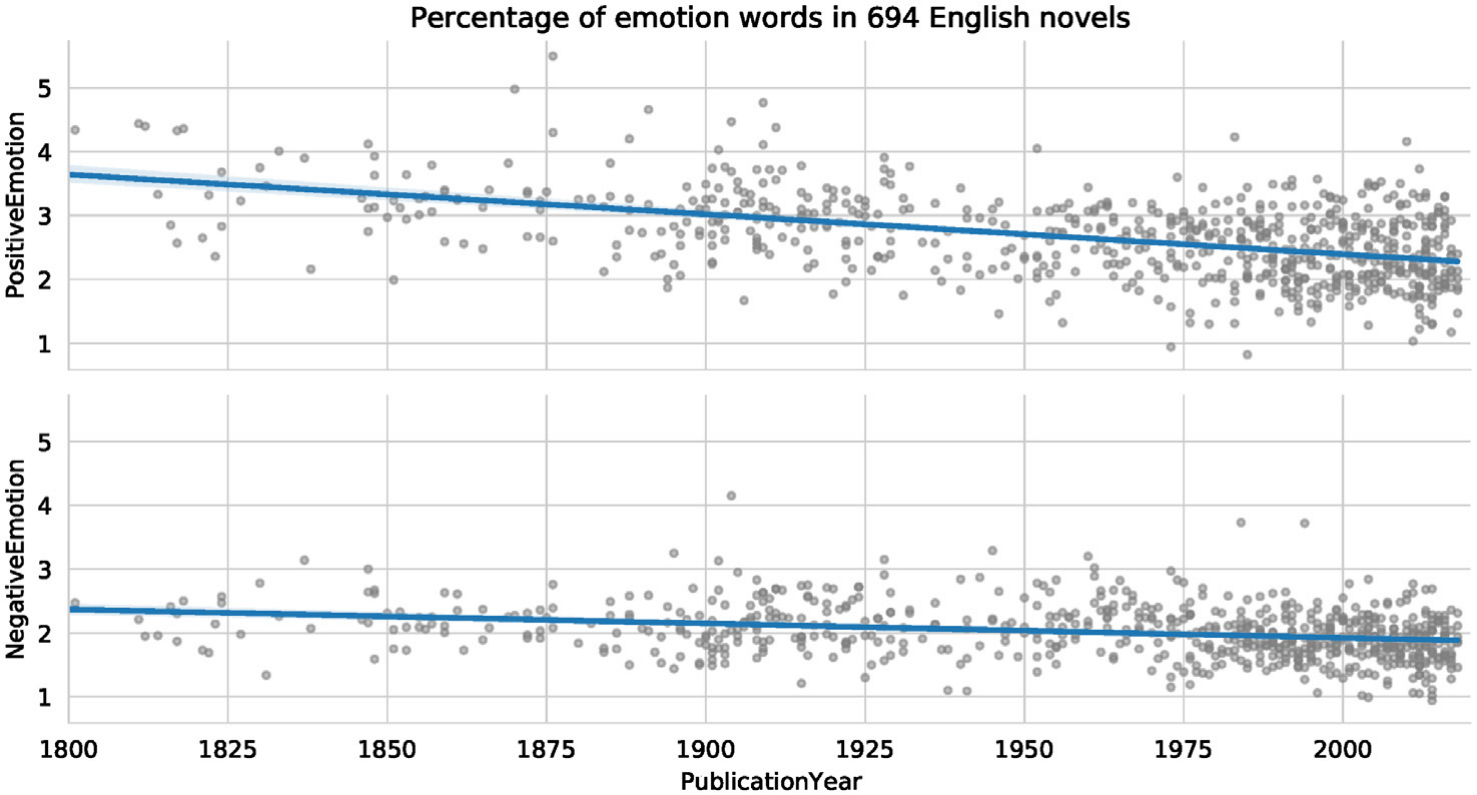
The percentage of emotion words in the English sample as a function of publication year.

Table 1 shows the central descriptive statistics of the English-language sample. Figs. 1–3 visualize, respectively, how the sample is comprised with regard to the authors’ nationality, publication year, and age at publication. Figs. 4–6 show the authors’ nationalities, publication year, and age at publication in the Dutch-language sample. Fig. 7 shows how the frequencies of positive emotion words and negative emotion words change as a function of publication year in the English-language sample.

**Table 1:** Descriptive statistics of the English-language sample (*n* = 694 novels, 66.9 million words).

	Heterosexual		Heterosexual		Homosexual		Homosexual		Bisexual	
	males		females		males		females		females	
	*M*	*SD*	*M*	*SD*	*M*	*SD*	*M*	*SD*	*M*	*SD*
Age	41.17	8.05	43.00	8.82	42.43	11.33	43.85	10.34	46.00	12.18
Publ. year	1942	58.65	1945	60.92	1975	38.60	1985	32.65	1935	65.63
Novels	151		153		167		158		65	
Authors	86		85		55		54		22	
Word count	16.8 million		15.9 million		15.7 million		13 million		5.5 million

## 2 Experimental Design, Materials and Methods

Electronic versions of the novels were downloaded from online sources and acquired from various other sources (see Specifications Table above). All novels were cleaned manually of prefaces, introductions, content tables, postscripts, biographical notes, author notes, footnotes, and publishers’ additional commercial material included at the end of many novels to prevent them from affecting the psycholinguistic analysis of the literary data. The processing of the Dutch novels was more involved since it included texts from different sources including printed books; this includes automatic processing steps such as normalizing punctuation to a basic set of punctuation characters and removing hyphenation. In the Dutch sets, scanned texts from offline sources were converted to text files using Optical Character Recognition (OCR) software, and manually corrected. The processing is further elaborated in appendix A of [20].^2^ The psycholinguistic data were then extracted from the text files using LIWC.

### 2.1 Psycholinguistic data

A commonly used method for linking language use with psychological variables involves calculating word frequencies based on manually created psycholinguistic categories of language [21,22]. Linguistic Inquiry and Word Count (LIWC) [16,17] is a popular tool for conducting these kinds of analyses. LIWC accesses either a single text file or a group of files and analyses each of them sequentially. Within each text file, LIWC reads one word at a time and compares it with the in-built dictionary file. If the target word is matched with a dictionary word, the appropriate word category (or categories) for that word is/are incremented. For each text file, LIWC assesses the relative frequency of approximately 93 linguistic and psycholinguistic output variables. This number has increased as the program has gone through revisions over the years, with the latest LIWC iteration published in 2015 [16,17]. The LIWC2015 data output is assorted into columns, which include total word count for each text file, four summary language variables (analytical thinking, clout, authenticity, and emotional tone),^3^ three general descriptor categories (words per sentence, percent of target words captured by the dictionary, and percent of words in the text that are longer than six letters), 21 standard linguistic dimensions (e.g., percentage of pronouns, articles, and verbs), 41 psychological construct categories (e.g., affect, cognition, biological processes, drives), six personal concern categories (e.g., work, home, leisure activities), five informal language markers (assents, fillers, swear words, netspeak, nonfluencies), and 12 punctuation categories (e.g., periods, commas, semicolons) [17]. The four summary variables (analytical thinking, clout, authenticity, and emotional tone) have values ranging from 0 to 100, which have been automatically converted by LIWC to percentiles based on standardised scores from large comparison samples [17]. The four summary variables are the only non-transparent dimensions in the LIWC2015 output: all the other LIWC variables are a percentage of total words in each category per text [17]. For details on the LIWC word categories, readers can refer to [17]. The Dutch-language data is derived using the validated Dutch translation of the 2001 version of LIWC [18]. LIWC2001 includes a more limited number of psycholinguistic categories than LIWC2015, totaling 68 categories.

### 2.2 Unigram and bigram counts

To derive unigram and bigram counts from the novels, the text files were preprocessed by converting them to lowercase and applying word tokenisation. Word tokenisation is the process of separating punctuation and words by identifying token boundaries. We used existing off-the-shelf tools for tokenisation.^4^ Contractions are represented as separate tokens (e.g., “can't” is rendered as “ca” “n't”). Each text is reduced to a bag of word counts, resulting in tables of counts with texts as rows and words as columns. We pruned the resulting document-term matrices in two ways: columns with occurrences in less than 10 texts were removed, and only the 100k most frequent features were retained. The absolute frequencies are reported. Using the provided overall counts with the sum of features across all texts, these can be converted to relative frequencies, z-scores, or tf-idf scores.

### 2.3 Limitations

The authors’ sexual orientation was determined based on biographical information, including information on the sex of any partners (married or otherwise) that the authors had or any self-identification related to sexual orientation that the authors may have made publicly known [e.g., 23,24,25,26,27,28]. This variable is therefore based on both manifest sexual behavior as well as self-identification; however, both sexual behavior and sexual orientation may undergo various changes over time, particularly in women [29,30], and therefore the use of an aggregate measure of lifetime sexual behavior and sexual orientation may not accurately track a person's sexual behavior or sexual orientation at any single point in time. Rather, this variable is used as an instructive overall indicator of an author's sexual behavior and attractions over their lifetimes, and as such may be limited by the availability of such information in biographical material.

## Supplementary Material

The data associated with this article can be found at http://dx.doi.org/10.17632/tmp32v54ss.2

## CRediT Author Statement

**Severi Luoto:** conceptualisation, methodology, software, validation, investigation, formal analyses, resources, data curation, project administration, writing: original draft preparation, writing: review & editing, visualization, project administration, and funding acquisition. S.L. collected the English-language data. **Andreas van Cranenburgh:** conceptualisation, methodology, software, validation, investigation, formal analyses, resources, data curation, project administration, writing: review & editing, visualization, and funding acquisition. A.v.C. collected the Dutch-language data. A.v.C. collated the unigram and bigram data for both English and Dutch samples.

## Declaration of Competing Interest

The authors declare that they have no conflicts of interest that could have influenced the contents of this article. The work was funded by Emil Aaltonen Foundation and The University of Auckland (S.L.), and by the Royal Netherlands Academy of Arts and Sciences through the Computational Humanities Program (A.v.C.).

## Footnotes

1 The only pre-19^th^ century novels were three novels by Aphra Behn published in the late 17^th^ century.

2 The processing code is available at https://gist.github.com/andreasvc/889c4acb3e9a77f44e91

3 *Analytical thinking*: this variable is a factor-analytically derived dimension based on eight function word dimensions. All eight function word categories load on a single dimension: two positively (articles, prepositions) and six negatively (personal pronouns, impersonal pronouns, auxiliary verbs, conjunctions, adverbs, and negations). A high value on this dimension reflects formal, logical, and hierarchical thinking; lower values reflect more informal, personal, here-and-now, and narrative thinking: see [31] for more details; *clout*: relative social status, confidence, or leadership displayed through language use; *authenticity*: language that indicates a speaker/writer who is more personal, humble, and vulnerable; *emotional tone*: a summary variable of the LIWC categories ‘positive emotion’ and ‘negative emotion’: the higher the number, the more positive the tone, with values below 50 suggesting a more negative emotional tone, see [17] for further details.

4 For English, we used the SynTok library: https://github.com/fnl/syntok For Dutch, we used the tokeniser that is part of the Alpino Parser: http://www.let.rug.nl/vannoord/alp/Alpino/
